# Supplementary material for: Animal Models of Drug-Resistant Epilepsy as Tools for Deciphering the Cellular and Molecular Mechanisms of Pharmacoresistance and Discovering More Effective Treatments
Source: Cells. 2023 Apr 24;12(9):1233. doi: 10.3390/cells12091233 (PMC10177106; doi:10.3390/cells12091233)
Supplement: Supplementary file 1 [file cells-12-01233-s001.zip › cells-2203018-supplementary.pdf]

Table S1. A comparison of anti-seizure potencies (ED_{50}s) of various anti-seizure medications (ASMs) in rodent models of drug resistant seizures and "minimal neurotoxicity" (TD_{50}s) in the rotarod test. See Figs. 2, 8, and 9 for illustration of these data. Sources are described in figure legends. Differences in TD_{50}s of the same ASMs in mice relate to the use of different mouse strains (see text).

	ED_{50} (mg/kg i.p.) or TD_{50} (mg/kg i.p.)											
ASM	Comparison of MES with amygdala kindled seizures in Wistar rats (Fig. 2)*					Comparison of MES with 6-Hz seizures in mice (Fig. 8)				Comparison of MES with SRS in intrahippocampal kainate model in mice (Fig. 9)		
	ED_{50} MES	ED_{50} fully kindled Wistar rats			TD_{50}	ED_{50} MES	ED_{50} 6-Hz		TD_{50}	ED_{50} MES	ED_{50} SRS	TD_{50}
		Focal seizures (EEG)	Focal seizures (stage 1-3)	Generalized convulsive seizures (stage 4-5)			32 mA	44 mA				
Carbamazepine	4	>20	15	8	37	9.8	17.9	16.5	58	9.8	84	45.4
Clonazepam	1.2	>2	>2	0.064	1.6	25.6	0.04		0.26			
Diazepam	5	>10	>10	1.4	2.8					23	1.5	4.7
Phenobarbital	12	>70	44	16	58	11.3	14.8	35.3	46	11.3	25	69
Phenytoin	14	50	50	30	140	6.7	>60	>60	51	6.7	>50	41
Primidone	17	>100	>100	>100	>40							
Valproate	100	300	220	190	275	263	126	310	398	263	280	398
Lamotrigine						5.4	>60	>60	30	5.4	>90	30
Lacosamide						4.5	20	15.2	27			
Levetiracetam						>500	19.4	1089	>500	>500	580	>500
Felbamate						35.5	70	241	220			
Pregabalin										11.6 (p.o.)	40	>300
Perampanel						1.6	2.1		1.8			
Retigabine						9.4	26	33	21			
Tiagabine						>5	0.66		1.29	>5	0.33	1.29
Topiramate						18.3	>300		234			
Vigabatrin										>2000	52	1570
Zonisamide						41	97		105			
Cenobamate						9.8	17.9	16.5	58			

*Group values without selection into responders and nonresponders
